# How emergency departments are responding to the opioid crisis: Results from a statewide survey in Kentucky

**DOI:** 10.1186/s13722-024-00512-3

**Published:** 2024-11-08

**Authors:** Olivia K. Sugarman, Samantha J. Harris, Lauren Byrne, Vivian Flanagan, Sabrina Gattine, Isha Desai, Omeid Heidari, Jason B. Gibbons, Sara Whaley, David Lucas, Tracy Pugh, Allison McBride, Brendan Saloner

**Affiliations:** 1 Department of Health Policy and Management, Johns Hopkins University Bloomberg School of Public Health, Baltimore, MD USA; 2 George Washington University Trachtenberg School of Public Policy and Public Administration, Washington, DC USA; 3 School of Nursing, Department of Child, Family, and Population Health Nursing, University of Washington, Seattle, WA USA; 4 University of Colorado Anschutz Medical Campus, Aurora, CO USA; 5 Vital Strategies, New York, NY USA

**Keywords:** Substance use disorder services in emergency departments, Medications for opioid use disorder, Bridge program, Needs assessment

## Abstract

**Objective:**

There is a rising effort for hospital emergency departments (EDs) to offer and expand substance use disorder (SUD) services. This state-wide evaluation studies SUD services offered along the continuum of implementation across Kentucky’s EDs to inform future state efforts to build ED bridge programs.

**Methods:**

We conducted a mixed-methods study using an online survey of all Kentucky Emergency Department Directors between January and May of 2023. We created a hospital-level dataset which we used to summarize quantitative questions and thematically analyze open-ended responses.

**Results:**

Our sample included 85 unique respondents (89% of all eligible Kentucky hospitals). Nine (11%) had active bridge programs to initiate opioid use disorder patients on buprenorphine. Respondents reported that the most challenging SUD-related services for EDs to implement were buprenorphine induction for opioid use disorder treatment (*n* = 36, 42%), referrals to community-based providers (*n* = 34, 40%), and providing social work services (*n* = 25, 29%). Respondents noted that the implementation and improvement of screening protocols were needed to better identify patients with SUD, expressed concerns about care continuity, and explicitly conveyed the need and desire for additional supports to provide SUD care.

**Conclusions:**

The landscape of Kentucky’s ED SUD supports shows several hospitals that offer services along the continuum of SUD care, and highlights the importance of technical assistance and financial resources to ensure the continuum is broadly available. Kentucky’s experience speaks to broader national challenges in supporting SUD in EDs – specifically the need for financial resources, buy-in and education, and creating referral relationships to ensure care continuity.

**Supplementary Information:**

The online version contains supplementary material available at 10.1186/s13722-024-00512-3.

## Background

As the opioid crisis continues in the United States, emergency departments (EDs) play an increasingly important role in providing both acute care for people who use drugs and as touchpoint to initiate longer-term substance use disorder (SUD) care and social supports [1–3]. Providing SUD supports like medications for opioid use disorder (MOUD) in the ED following a non-fatal opioid overdose significantly decreases the likelihood of subsequent negative health outcomes, but many patients do not receive SUD care following an overdose [4, 5]. Without intervention, overdose survivors have an increased risk of subsequent overdoses [6–8] and people who experience a non-fatal overdose are three times more likely to die by fatal overdose [9]. 

While more EDs are adopting SUD supports, screening and treatment are not the standard of care for SUD patients who present in EDs. Existing standards of care encourage, but do not require that EDs offer SUD supports; patients are instead treated for acute needs and discharged once stable [1]. One innovative model to integrate SUD supports in EDs are bridge programs. ED bridge programs integrate low-threshold SUD care into hospital settings for patients who are admitted to EDs for drug-related health events [2, 10]. Bridge clinic supports can include SUD screening and diagnosis, connections to social services, peer supports, and harm reduction supplies, timely patient access to MOUD, and warm handoffs to community providers and resources [2, 10]. 

Following the success of bridge programs in other states and in response to high overdose mortality in Kentucky [2, 11, 12], the Kentucky Statewide Opioid Stewardship (KY SOS) program has sought to support existing programs and increase the number of bridge programs throughout the state modeled after efforts in other states such as California [10, 13, 14]. KY SOS is an initiative comprised of leaders from the Kentucky Cabinet for Health and Family Services’ Kentucky Opioid Response Effort and the Kentucky Hospital Association that aims to support hospitals in their commitment to combatting the state’s opioid epidemic by providing technical assistance and additional resources for member hospital staff and leadership [15]. 

While there are increasing efforts across the nation to encourage bridge clinic adoption, there is a lack of comprehensive statewide data on how hospitals are approaching implementation of SUD supports. Baseline assessments of existing SUD supports in hospitals and EDs are important as state and federal policies and programs evolve to support and codify hospital SUD care minimums. Where prior literature focuses on implementation of ED SUD programs in select vanguard hospitals [16–19], this study provides state-wide evidence of SUD-related care and barriers along the continuum of implementation. To inform Kentucky’s efforts, we conducted a state-wide baseline assessment to understand the existing breadth, capacity, and challenges of offering hospital-based SUD supports in Kentucky, and potential supports needed to implement bridge programs. In this mixed-methods study, we surveyed Kentucky hospitals and examined existing (a) hospital-level SUD services offered in EDs, (b) referral practices, (c) perceived barriers and facilitators, and (d) overall priorities and challenges to offering SUD-related services in individual EDs.

## Methods

### Study design

We designed a mixed-methods cross-sectional survey to examine current Kentucky hospital ED practices in supporting patients with SUD. The survey instrument included closed-ended multiple-choice and open-ended response questions. The survey was distributed electronically via Qualtrics to an email listserv of 96 ED Directors throughout the state, including ED Directors at nine hospitals with established bridge programs. ED Directors were chosen as primary contacts because they were likely to be the most knowledgeable about hospital and ED protocols to treat patients who present with SUD-related needs. Survey responses were collected between January 26, 2023, and May 15, 2023. Follow-up with potential respondents continued until 89% completion (*n* = 85) was reached. Respondents were not compensated for participation.

The survey instrument was adapted from the literature and previous surveys of hospitals in Michigan and Pennsylvania that similarly aimed to assess statewide hospital and ED capacity to serve people with substance use-related needs [20, 21]. Survey content was collaboratively tailored with representatives from the Kentucky Hospital Association and Kentucky Opioid Response Effort. We examined Kentucky hospital ED supports for patients with SUD across several key survey domains, including: hospital and ED pharmacy characteristics, SUD screening protocols, buprenorphine prescribing protocols, referrals to community providers, and overall perceived priorities and challenges to implement SUD supports in EDs (Table 1). The complete survey is provided in Appendix 1.

**Table 1:** Survey domains and exemplary survey questions

Domain	Sample Survey Questions
Hospital characteristics and pharmacy access	• Is buprenorphine on your hospital’s formulary? • Does your ED have a Pixys Medstation system?
Screening	• Does your ED have a screening protocol (e.g., SBIRT) to identify whether a patient has a substance use disorder? • Is the screening protocol built into the electronic health record system used by the ED? • Which patient populations are screened for SUD?
X-waivered providers and the 72 h rule^a^	• Does your ED have a protocol to prescribe buprenorphine to patients with opioid use disorder? • Does the ED have physicians, PAs, or APRNs who dispense buprenorphine using the 72-hour rule instead of under an X-waiver? • How many providers in your ED have an X-waiver to prescribe buprenorphine? • Does the ED facilitate a home induction option, where providers with an X-waiver prescribe buprenorphine for patients to take home once their opioid withdrawal is sufficient?
Counseling & education	• Does the ED provide any non-medication interventions for patients with OUD (e.g., counseling, motivational interviewing)? • Are any non-medication interventions required for patients prior to receiving MOUD?
Referrals to community partners	• Is there an existing bridge clinic in or near the hospital that the ED connects patients do? • What are the barriers to making warm handoffs to community providers?
Peer support services	• Does the hospital utilize peer support specialists or coaches in the ED? • When are peer support specialists available?
Social services	• Does the hospital provide social services or referrals to social services for patients with SUD? Who provides these services?
Harm reduction	• Does the hospital provide harm reduction services or referrals to harm reduction services for patients with SUD? Who provides these services? • Does your ED have a Naloxone take home protocol?
Training	• Does your ED have plans to implement stigma reduction training for ED staff? • Does your ED have plans to implement a racial equity training for ED staff?

^a^The X-waiver requirement was removed while the survey was being fielded

We created a hospital-level dataset after surveys were completed. Responses were included in the final analysis if the respondent consented to participate and completed the survey in its entirety. In some cases, more than one respondent completed a survey on behalf of the same hospital. Of hospitals with more than one recorded survey response, we included only the most recent response in the analysis. The most recent respondent was selected because we assumed (1) the response would reflect the most current practices, and (2) if the survey was initially sent to a person without relevant knowledge about the hospital’s ED SUD offerings, the final respondent most likely received the survey from prior, less knowledgeable respondents. Seven of 96 possible respondent hospitals had more than one respondent. Six hospitals had two respondents, one hospital had three respondents.

### Analysis

We summarized and tabulated response frequencies of closed-ended multiple-choice survey questions across all hospitals and domains. Some questions were only available to some respondents based on their previous answers (i.e. because of programmed survey logic), so denominators may differ for some survey questions. For open-response questions, two study team members (OS, SH) compiled and reviewed individual responses for thematic trends and exemplary quotes along the key survey domains. Analysts independently reviewed all open responses, then compared findings for each open response until agreement was achieved. This study was approved by the Institutional Review Board at Johns Hopkins Bloomberg School of Public Health. Analysis was completed using SAS software 9.4.

## Results

### Quantitative results

Final analysis included 85 unique responses, representing 85 of 96 possible respondent hospitals and a response rate of 89%. Survey domains and exemplary questions are provided in Table 1.

#### Existing protocols and services

Table 2 reports existing hospital protocols for substance use and harm reduction-related services offered in EDs in our study population. While most hospitals in our sample had buprenorphine on their formulary (*n* = 67, 79%), few EDs had their own pharmacy that could also dispense buprenorphine (*n* = 6, 7%). Alternatively, some EDs used Pixys Medstations (*n* = 74, 87%), a secure, mobile, automatic medication dispensing machine. Among EDs with Pixys Medstations, 38% stocked buprenorphine (*n* = 28 of 74). Nearly 40% of hospitals (*n* = 33) had an outpatient pharmacy with buprenorphine that patients could use to access longer-term refills (greater than 3 days) of buprenorphine.

**Table 2:** Kentucky hospital and emergency department substance use disorder services offered

Variable	Yes		No		Do not know	
	*n*	%	*n*	%	*n*	%
**Pharmacy**						
Buprenorphine on hospital pharmacy’s formulary	67	79%	14	16%	4	5%
Hospital has outpatient pharmacy with buprenorphine	33	39%	48	56%	4	5%
ED has buprenorphine in Pixys medstation (*n* = 74)^a^	28	38%	43	58%	3	4%
ED has own pharmacy	6	7%	79	93%	0	0%
**SUD Screening**						
ED has screening protocol to identify patients with substance use disorder	37	44%	43	51%	5	6%
Protocol built into EHR (*n* = 37)^b^	36	97%	1	3%	0	0%
Which patients are screened for SUD (*n* = 37)^b^						
All	25	68%	-	-	-	-
Some	11	30%	-	-	-	-
None	1	3%	-	-	-	-
**MOUD Prescribing**						
ED has written protocol to prescribe buprenorphine to patients with OUD	9	11%	70	82%	6	7%
ED facilitates home buprenorphine induction	2	2%	64	75%	19	22%
**Approximate percentage of ED providers who prescribed buprenorphine in past 12 months**						
None	48	56%	-	-	-	-
1 − 25%	7	8%	-	-	-	-
26 − 50%	2	2%	-	-	-	-
51 − 75%	1	1%	-	-	-	-
Over 75%	1	1%	-	-	-	-
Do not know	26	31%	-	-	-	-
Require counseling, behavioral health intervention to receive MOUD	7	8%	52	61%	26	31%
**Peer supports and social services**						
Hospital utilizes peer supports in ED for patients with SUD	11	13%	68	80%	6	7%
ED has direct access to hospital and/or ED social workers for patients with substance use disorder	42	49%	37	44%	-	-
**Harm reduction standard protocol services** ^**c**^						
Overdose education	46	54%	-	-	-	-
Safer use education	28	33%	-	-	-	-
No harm reduction services provided	20	24%	-	-	-	-
Take-home naloxone	20	24%	-	-	-	-
Other (please specify)	10	12%	-	-	-	-
Co-prescribing naloxone with opioid prescriptions	9	11%	-	-	-	-
Safer use supplies such as fentanyl test strips	0	0%	-	-	-	-
Wound care kit	0	0%	-	-	-	-

^a^Of hospitals with Pixys Medstations (*n* = 74)

^b^Of hospitals with an SUD screening protocol (*n* = 37)

^c^Select all that apply question, percentages may exceed 100%

Less than half of EDs reported having SUD screening protocols (*n* = 37, 44%). Of those with screening programs, almost all (*n* = 36 of 37, 97%) had their screening protocols programmed into EHRs. When asked about specific practices for SUD screening, the majority, 68% (*n* = 25) of the 37 hospitals screened all patients for SUD, 30% (*n* = 11) screened only some based on certain patient characteristics (e.g. have a Hepatitis C diagnosis, opioid prescription, present with overdose).

Buprenorphine prescribing protocols and prescriber capacity were also limited. Nine respondents (the nine established bridge clinics; 11% of all respondents) each had a written protocol to prescribe buprenorphine to patients who screened positive for OUD. Among all hospitals, only two (2%) facilitated home induction. Most respondents reported that there were no ED providers who prescribed buprenorphine in the ED in the previous 12 months (*n* = 48, 56%), or that they did not know (*n* = 26, 31%). Seven EDs (8%) required patients to seek counseling or another behavioral health intervention in order to receive MOUD. The minority used peer supports in the ED for patients with SUD (*n* = 11, 13%), though almost half had direct access to hospital and/or ED-based social workers for patients with SUD (*n* = 41, 49%). Most EDs offered at least some harm reduction services, with education or naloxone provision as the most common, though still a quarter (*n* = 20, 24%) offered no harm reduction services.

### Barriers and facilitators to offering SUD services

Table 3 describes barriers and facilitators for EDs to offer specific SUD services, including pharmacy buprenorphine availability, screening for SUD, and prescribing buprenorphine.

**Table 3:** Barriers and facilitators to offering specific substance use disorder-related services

Variable	*n*	%
**Screening**		
**What barriers does your ED face related to screening patients for substance use disorder? ** ^a^		
Need to triage competing medical problems	36	42%
Lack of clinical knowledge/training in administering substance use disorder screening	33	39%
Screening patients for substance use disorder is not part of the ED protocol	32	38%
Lack of adequate substance use disorder screening tools	30	35%
Screening is not embedded within the EMR	30	35%
Lack of time	25	29%
Lack of training in what to do with a positive screen	25	29%
Nowhere to refer patients with a positive screen	23	27%
Patient privacy concerns (e.g., family member or significant other will not leave the room)	23	27%
Some staff are uncomfortable screening patients for substance use disorder	12	14%
Other	10	12%
***What factors make it easier to screen patients for substance use disorder? ***(*n* = ***37)***^***a, b***^		
Substance use disorder screening is embedded in the EMR	31	84%
Substance use disorder screening is part of the ED protocol	18	49%
Providers have clinical knowledge/training in administering substance use disorder screening	12	32%
Providers know how/where to refer patients with a positive screen	11	30%
Providers are comfortable administering substance use disorder screenings	10	27%
Providers are trained in what to do with a positive screen	9	24%
ED has a champion who has led education efforts about screening for substance use disorder	4	11%
Other	2	5%
**MOUD**		
**Rank your level of agreement with the following statement: Patients with opioid use disorder in the ED can receive buprenorphine in a timely manner.**		
Strongly disagree	6	7%
Disagree	6	7%
Neither agree nor disagree	24	28%
Agree	26	31%
Strongly agree	13	15%
Not Applicable	10	12%
**What barriers are there to prescribing take-home buprenorphine?** ^a^		
Lack of providers that have/had an X-waiver to prescribe buprenorphine ^c^	56	66%
Lack of clinician willingness to prescribe buprenorphine	44	52%
Lack of clinician knowledge in how to induct patients on buprenorphine	41	48%
Clinicians often will not prescribe buprenorphine unless patients are connected to counseling or treatment	36	42%
Lack of knowledge that patients can receive take-home buprenorphine from X-waivered providers	30	35%
No community providers to continue prescriptions after take-home supply runs out	29	34%
Lack of time to follow up with patient when they leave the ED	28	33%
Pharmacy does not stock buprenorphine or maintain adequate supplies	21	25%
Lack of patient interest	9	11%
Limited access to pharmacy or long wait times	9	11%
Other	8	9%

^a^Select all that apply question, percentages may exceed 100%

^b^Only among hospitals with a screening protocol

^c^The X-waiver requirement was removed while the survey was being fielded

**Screening.** Triaging competing medical problems was the top barrier to treating people with SUD in the ED for 42% (*n* = 36) of hospitals. About 40% of respondents (*n* = 33) said ED providers had a lack of clinical knowledge or training in administering SUD screenings, and 38% (*n* = 32) said screening patients for SUD was not part of the ED protocol. About a quarter said lack of time and lack of training in what to do with positive screens (both *n* = 25, 29%), referral resources, and patient privacy concerns were barriers to SUD screening (both *n* = 23, 27%). Of participants who responded with “other”, common screening barriers were provider attitudes, patients leaving against medical advice, or patients withholding information due to stigma. One said patients with SUD only receive resources if they have a co-occurring behavioral health issue.

Of the 37 hospitals that screened for SUD, embedding the screening tool in EHRs was the most common facilitator to SUD screening (*n* = 31 of 37, 84%). ED health providers’ clinical knowledge (*n* = 12 of 37, 32%), comfort (*n* = 10 of 37, 27%) in administering SUD screening tools, and where to make referrals (*n* = 11 of 37, 30%) were other common facilitators of SUD screening. One respondent noted that having a behavioral health team in the ED 24/7 facilitated the screening process. Another said peer supports in the ED were helpful.

**MOUD prescribing.** Respondents generally agreed that patients with OUD in the ED could receive buprenorphine in a timely manner (agree, *n* = 39, 46%; neither disagree or agree, *n* = 24, 28%, disagree, *n* = 12, 14%; not applicable, *n* = 10, 12%). However, about half said that clinicians didn’t know how to induct patients on buprenorphine (*n* = 41, 48%), and more than half of respondents indicated that clinicians were not willing to prescribe buprenorphine (*n* = 44, 52%). Many respondents (*n* = 36, 42%) said clinicians would not prescribe buprenorphine unless patients were going to be connected to follow-up counseling or treatment. Similarly, a third of respondents said a barrier to buprenorphine prescribing was the lack of community providers to continue prescriptions after take-home supplies run out (*n* = 29, 34%). A quarter of respondents indicated “other” barriers to get ED providers to obtain an X-waiver, such as a lack of encouragement to prescribe buprenorphine.

### Referrals

**SUD treatment referrals.** Table 4 describes referral processes and barriers and facilitators to making referrals for substance use-related supports after discharge. The most common services EDs referred to were behavioral health providers (*n* = 57, 67%), primary care providers (*n* = 48, 56%), and outpatient or inpatient substance use treatment (outpatient, *n* = 42, 49%; inpatient, *n* = 35, 41%). Only nine reported having no referral process in place (*n* = 9, 11%).

**Table 4:** Hospital referral services, barriers and facilitators to offering referrals for substance use disorder services

Variable	*n*	%
**Presence of an existing bridge clinic in or near the hospital that the ED uses to refer patients**		
Yes	25	29%
No	44	52%
Do not know	16	19%
**Services ED refers to**:		
Behavioral health provider (e.g., psychiatrist, licensed counselor, etc.)	57	67%
Primary care provider	48	56%
Outpatient substance use treatment	42	49%
Inpatient substance use treatment	35	41%
Residential substance use treatment	19	22%
Buprenorphine treatment	18	21%
Opioid treatment program (i.e., methadone treatment)	12	14%
No referrals process in place	9	11%
Other	8	9%
Step down care within the hospital	6	7%
**What are the top barriers to making warm hand-offs to other providers for follow-up care?** ^**a**^		
Our ED currently does not have a protocol for referrals	42	49%
Lack of staff who can coordinate hand-offs	38	45%
No partnerships with existing providers	32	38%
Lack of time to coordinate	30	35%
No providers nearby	19	22%
Patients not interested in handoffs	17	20%
Providers do not want to onboard patients who are in crisis	11	13%
Other	11	13%
No providers with availability for new patients	6	7%
No providers who accept Medicaid as payment	1	1%
**Harm reduction**		
**Which of the following harm reduction services does your ED refer to community partners?** ^**a**^		
No referrals are made for harm reduction	40	47%
Local health department (safer use discussion/education, wound care kit, etc.)	32	38%
Take-home naloxone (community RX, mobile naloxone unit, etc.)	21	25%
Syringe access services (needle exchange program)	18	21%
Other	6	7%
**Social Services**		
**Which of the following social services does the** **ED refer patients** **with substance use disorder?** ^**a**^		
Assistance obtaining Medicaid or other health coverage	35	41%
Assistance with transportation	31	36%
Housing resources	26	31%
None of the above	26	31%
Assistance navigating insurance benefits	19	22%
Documentation (e.g. ID card)	17	20%
Other	13	15%
**What barriers does the ED face in providing services or referrals for social services?** ^**a**^		
ED does not have the capacity to contact patients after discharge to ensure care continuity	52	61%
Lack of partnerships with existing service providers	39	46%
Lack of service providers nearby	41	48%
Lack of service providers with availability for new clients	26	31%
Lack of staff to coordinate services or referrals	43	51%
Lack of time to coordinate services or referrals	36	42%
Patients are not interested in services or referrals	30	35%
Other	10	12%
**What factors help your ED provide services or referrals for social services?** ^**a**^		
ED has a champion for connections to social services	6	7%
ED has existing partnerships with social service providers	15	18%
ED has follow-up care staff who contact patients after discharge to ensure care continuity	11	13%
ED has social services navigators on staff	17	20%
Other	52	61%

^a^Select all that apply question, percentages may exceed 100%

The most common barriers to facilitating referrals to outpatient SUD providers for follow-up care in the community, in rank order, were that the ED did not have a protocol for referrals (*n* = 42, 49%), lacked staff to coordinate handoffs (*n* = 38, 45%), and lacked partnerships with existing providers (*n* = 32, 38%). Almost a quarter of respondents (*n* = 19, 22%) indicated there were no providers nearby to refer to.

**Harm reduction referrals**. Table 4 also describes ED referrals for harm reduction and social services. While less than half of EDs reported they made referrals for harm reduction services (*n* = 40, 47%), those who did made such referrals to local health departments (*n* = 32, 38%) and syringe services programs (*n* = 18, 21%). Only a quarter offered referrals for take-home naloxone (*n* = 21, 25%).

**Social services referrals.** About 70% of EDs reported that they referred patients with SUD to social services (*n* = 26, 31% did not offer services). Referred social services included Medicaid or other insurance enrollment assistance (*n* = 35, 41%), assistance with transportation (*n* = 31, 36%), and housing resources (*n* = 26, 31%). One “other” service referral mentioned was by an ED that provided patients with crisis phone lines to call.

**Barriers and facilitators to offering referrals to social services.** The most common barrier to referring patients to social services was a general lack of capacity to contact patients after discharge to ensure care continuity (*n* = 52, 61%). Similarly, over half of respondents said their ED lacked staff to coordinate referrals (*n* = 43, 51%). Others cited a general lack of nearby providers to refer to (*n* = 39, 46%), or lack of service providers with availability for new clients (*n* = 26, 31%). Roughly a third of respondents said patients were simply not interested in receiving social services or referrals (*n* = 30, 35%).

Few hospitals said that their ED had social services navigators on staff (*n* = 17, 20%), had existing partnerships with social service providers (*n* = 15, 18%), had follow-up care staff who could contact patients after discharge (*n* = 11, 13%), or had a champion for connections to social services (*n* = 6, 7%). Several respondents provided additional information about social worker availability in the ED (e.g., several had ED social workers on site or on call 24/7, while others have hospital social workers available during normal business hours).

### Training

Table 5 reports awareness of staff participation in trainings relating to stigma and racial equity. When asked about awareness of staff participation in stigma reduction trainings, around a quarter said staff had participated (*n* = 20, 24%), 46% (*n* = 38) said staff had not participated in such trainings, and a third of respondents did not know (*n* = 24, 29%). Over half (*n* = 45, 55%) did not know whether their ED had plans to implement or expand stigma reduction training. Most participants (*n* = 60, 73%) endorsed that staff had participated in cultural competency training, often noting it was a requirement in annual trainings.

**Table 5:** Stigma and racial bias training for ED staff

Variable	*n*	%
Have any of the ED staff participated in any form of stigma reduction training (training focused on reducing negative attitudes towards individuals with substance use disorder)?		
Yes	20	24%
No	38	46%
Don’t know	24	29%
Does your ED have plans to implement or expand stigma reduction training for ED staff?		
Yes	6	7%
No	31	38%
Don’t know	45	55%
Have any of the ED staff participated in any form of racial equity or cultural competency training (training focused on addressing and reducing the negative attitudes and implicit bias towards Black, Indigenous, and People of Color (BIPOC) individuals)?		
Yes	60	73%
No	10	12%
Don’t know	12	15%
Does your ED have plans to implement or expand racial equity training for ED staff?		
Yes	16	20%
No	25	30%
Don’t know	41	50%

### Most challenging and important SUD services to implement in EDs

Perceptions of the most challenging and most important SUD services to implement in EDs were consistent with findings in other domains (Table 6). Offering referrals to community-based providers was reported as both a top challenge (*n* = 34, 40%) and viewed as one of the most important (*n* = 32, 38%) services to implement. The top cited challenging SUD-related service for EDs to implement was buprenorphine induction (*n* = 36, 42%), though inducting patients on buprenorphine (*n* = 14, 16%) was viewed as less important to implement than most other services including screening (*n* = 32, 38%), referrals to community-based providers (*n* = 32, 38%), counseling and education (*n* = 22, 26%), providing social work services (*n* = 22, 26%), or providing naloxone (*n* = 15, 18%).

**Table 6:** Top two perceived most challenging, important substance use services for EDs to implement

Variable	*n*	%
**Top two perceived most challenging services for EDs to implement** ^a^		
Buprenorphine induction in the ED	36	42%
Referring to community-based providers	34	40%
Social work services	25	29%
Increasing x-waivered providers^b^	23	27%
Peer support specialists	18	21%
Naloxone dispensing	17	20%
Screening for substance use disorder	14	16%
Counseling and education	8	9%
ED staff stigma reduction	6	7%
Harm reduction	3	4%
Other (please specify)	1	1%
**Top two perceived most important services for EDs to implement** ^a^		
Screening for substance use disorder	32	38%
Referring to community-based providers	32	38%
Counseling and education	22	26%
Social work services	22	26%
Naloxone dispensing	15	18%
Buprenorphine induction in the ED	14	16%
Peer support specialists	11	13%
Harm reduction	9	11%
ED staff stigma reduction	7	8%
Increasing x-waivered providers^b^	5	6%
Other	2	2%

^a^Select all that apply question, percentages may exceed 100%

^b^The X-waiver requirement was removed while the survey was being fielded

The other perceived most important services for EDs to implement, in addition to community-based provider referrals (*n* = 32, 38%), were screening for SUDs (*n* = 32, 38%), counseling/education (*n* = 22, 26%), and social work services (*n* = 22, 26%). Providing social work services, however, was viewed as one of the most challenging (*n* = 25, 29%) services to implement, likely due to limited availability of social workers in the ED. Respondents did not view screening (*n* = 14, 16%) or counseling/education (*n* = 8, 9%) to be as challenging to provide as most other services.

### Qualitative results

Table 7 provides exemplary quotes along our survey domains that triangulate quantitative findings. We report key qualitative themes that emerged in our analysis below.

**Table 7:** Survey domains, qualitative themes, and Exemplary quotes documenting facilitators and barriers to providing SUD services

Theme	Quotes
Perceptions of bridge program need/Readiness to implement	*“Our volume of patients who have an opioid addiction have decreased drastically ever since they know that most of our physicians don’t prescribe narcotics and refer them to their primary care physician if needed.”* *“To even think an ED should be the starting place for opioid dependence treatment is poor judgment. Establish a state funded mental health and drug recovery system that is freely and immediately available to every patient referred by any Doctor or ACP.”* *“Staff buy-in of importance and significance of [the] problem is key. To understand the scope of the problem with our patients*,* we have to know the ‘who’ and ‘how much.’ To get this [bridge program] started and have staff and patients understand why we are asking will be challenging. Like growing pains.”* *“Funding for Naloxone and Peer Support Specialists is critical.”* *“Any toolkits or knowledge that can be shared on how to initiate/implement these programs into the ED [would be helpful].”* *“There is much desire to improve to the care we give to patients with OUD. Having resources and successful treatment pathways to model would be very helpful. Outpatient resources are a big knowledge gap.”* *“This program will be a burden on critical access hospitals. However*,* we are not strictly opposed to participating with adequate resources*,* training and consultation.”* *“I am very open to any additional support*,* services*,* educational materials*,* or plans for implementation as this is already begun as a passion project in our department.”*
Availability of buprenorphine in the ED	*“Buprenorphine induction is challenging when the hospital pharmacy is closed and the lack of a specific pathway for a community provider or service to hand off further care for the patient makes it difficult to coordinate the care of the patient and ensure continued MAOT (medication for alcohol and opioid treatment) after they leave the ED.”* *“Hospital leadership has been opposed to Suboxone inclusion in the formulary since inception due to stigma.”* *“Inventory of buprenorphine is difficult to manage in a small facility with few providers.”*
Barriers and facilitators to SUD screening	*“Lack of provider education on how to treat positive COWS scores. RN will page MD but no orders will be initiated.”* *“Behavioral Health Team in ED 24/7.”* *“If they are here for a drug issue only*,* without a psych component…we lack resources for them.”* “*Providers are not on board.”*
Barriers and facilitators to MOUD prescribing – community referrals	*“Buprenorphine induction is not always done because of how difficult it can be to hand off patients once they leave the ED.”* *“Resources are provided*,* but patients are not officially referred.”* *“Our facility does not see many patients seeking this type of treatment*,* and our providers are not comfortable prescribing the medications to treat it. This is mostly due to a lack of ability to follow these patients. I do not think this is a treatment that ED should provide. It is very difficult to follow up and keep in contact with patients from an ED standpoint. We struggle to get in contact with patients for treating medical conditions after ED visits. Opioid use disorder patients should be encouraged to use the outpatient resources available to them and maintain treatment within that environment. ED should be used to treat emergency conditions that may arise surrounding that outpatient treatment only.”* *“We are a small rural facility with limited community resources*.” *“If our staff had education*,* we could make the appropriate referrals.”*
Barriers and facilitators to MOUD prescribing – fear of legal repercussion & stigma	*“I am terrified of the legal liabilities to the ED physician if these meds are prescribed and negative outcomes occur. There is no outpatient service/system to guarantee proper follow up and coordinated care.”* *“There is a huge concern for liability without those concerns addressed in very specific ways*,* I WILL NOT initiate any outpatient dependence care.”* *“Leadership and community related stigma [is a barrier to prescribing buprenorphine].”*
Availability of peer support specialists	*“Having a network of providers/provider groups with reliable availability to take care of the patients once treated in the ED is a major barrier to successful MAOT (medication for alcohol and opioid treatment) initiation. Peer support specialists would help assist in keeping patients on track to continue MAOT and have support after they leave the ED. This is especially important in the timeframe between discharge and seeing their next provider outpatient.”* *“Peer support in the ER is a very beneficial asset. I have seen it benefit so many individuals when having someone to talk to in person at the moment of crisis.”* *“We don’t have peer support specialists in rural communities.”*
Availability of social workers to assist in making social service referrals	*“Presently we have no social workers employed in the inpatient or ER setting of our facility.”* *“Our social workers primarily care for the Behavioral Health unit patients. Our case workers help coordinate transfers but only pamphlet help for substance abuse disorders.”* *“We can contact that hospital social worker team during normal business hours or the on-call team after hours for help in referring patients.”* *“[We] provide patients with a crisis line to call for additional resources.”* *“Due to our hospitals size we have limited social work services that are always needed.”*
Availability of harm reduction services (e.g., naloxone)	*“Narcan and syringes [are] not available in this county [to refer to].”* *“Co-prescribing naloxone is our best practice.”* *“In discussions for system wide protocol through vending machines.”*
Barriers to reducing stigma and racial bias	*“Need more education and resources for staff to increase their level of competence and overall comfort for this problem.”* *“Race aside*,* there is no interest by physicians to prescribe any medications for outpatient opioid use reduction.”* *“Lack of translators*,* social workers*,* and care managers to get marginalized populations into detox programs or follow up care.”*

### Perceptions of bridge program need and implementation readiness varied

Perceptions of bridge programs and states of implementation were mixed. Some had well established programs or were interested in expanding SUD services. One said, “There is much desire to improve to the care we give to patients with OUD.” A few did not recognize a need for or were not in favor of offering SUD services. One expressed perceived decreases in patients with SUD receiving care in their ED due to the EDs lack of prescribing controlled substances, saying, “Our volume of patients who have an opioid addiction have decreased drastically ever since they know that most of our physicians don’t prescribe narcotics.” Another believed the ED was not the appropriate venue to provide SUD services at all, stating, “To even think an ED should be the starting place for opioid dependence treatment is poor judgment.”

In anticipation of planning to implement a bridge program, one participant summarized sentiments expressed by several, noting that adequate detection of SUD and staff and patient education would be needed, and recognizing it would take time and there would be “growing pains.” Several supported implementation and requested resources such as implementation toolkits and education opportunities. As one said, “I am very open to any additional support, services, educational materials, or plans for implementation as this is already begun as a passion project in our department.” Another shared, “There is much desire to improve the care we give to patients with OUD. Having resources and successful treatment pathways to model would be very helpful. Outpatient resources are a big knowledge gap.” Others said funding for naloxone and peer support specialists would be helpful.

### Referral barriers, stigma, and legal concerns inhibited willingness to prescribe buprenorphine

A lack of community providers to refer patients to was a key barrier to providing SUD services. Concerns about limited referral options often inhibited buprenorphine prescribing, as one said, “Buprenorphine induction is not always done because of how difficult it can be to hand off patients once they leave the ED.” Some expressed the need for additional education, with one saying, “If our staff had education, we could make the appropriate referrals.” Another said, “Our providers are not comfortable prescribing the medications to treat it. This is mostly due to a lack of ability to follow these patients. I do not think this is a treatment that ED should provide.” One attributed the lack of buprenorphine availability in their ED to stigma, saying, “Hospital leadership has been opposed to Suboxone inclusion in the formulary since inception due to stigma.”

Some feared potential legal repercussion from potential adverse health outcomes and/or diversion if buprenorphine was prescribed out of the ED, especially without referrals to community-based care. As one said, “I am terrified of the legal liabilities to the ED physician if these meds are prescribed and negative outcomes occur. There is no outpatient service/system to guarantee proper follow up and coordinated care.” Follow-up care was seen as especially challenging in rural areas, as one respondent said they worked in a “small rural facility with limited community resources.”

### Many faced resource constraints that limited perceived ability to provide SUD services

Several respondents noted challenges with implementing SUD services due to resource constraints such as staffing and hospital size. As one said, “Inventory of buprenorphine is difficult to manage in a small facility with few providers.” Social workers and peer support specialists were also not commonly available, with one saying, “Due to our hospitals size we have limited social work services that are always needed.” Another said, “We don’t have peer support specialists in rural communities.” Regarding barriers to reducing stigma and unconscious bias, one said their ED has a “Lack of translators, social workers, and care managers to get marginalized populations into detox programs or follow up care.” One said “staff burnout” made stigma reduction in the ED one of the most challenging issues to address.

## Discussion

We surveyed Kentucky hospital ED directors to examine existing ED SUD services, referral practices, and priorities and challenges to offering SUD services in Kentucky hospital EDs. Buprenorphine stocking in hospital or ED pharmacies was relatively low, only some hospitals had an SUD screening protocol, and continuity of care via referrals to outpatient care was a commonly cited challenge. Across domains, respondents expressed concerns about the lack of handoffs for care continuity, including for community provider referrals for MOUD, harm reduction, and social services. Perceptions of the utility of buprenorphine prescribing and bridge programs were mixed, but many supported peer support services and bridge clinic implementation and requested additional resources to support implementation. Three primary challenges in offering SUD services in EDs were: lack of SUD screening, limited buprenorphine availability, and lack of ability to make referrals to community providers.

Our study examines the full spectrum of adoption of ED SUD supports in a state that continues to be hard hit by the overdose crisis [12, 22]. For the past decade, Kentucky has been among the top ten states with the highest death rates. In 2022, Kentucky’s death rate was 53.2 per 100,000 compared to the U.S. average of 32.6 per 100,000 in the same year [12, 23]. Altogether, findings revealed gaps in the availability of SUD care in hospital EDs, but growing interest in offering peer support and SUD screening with additional funding and technical assistance.

SUD screening was one pervasive challenge for EDs, as less than half of hospitals had an SUD screening protocol. SUD was not a primary concern in some communities, so screening for SUD competed with triaging other health needs, and many respondents reported providers were not trained to screen for SUD. Several respondents noted reluctance to screen for SUD because of concerns they had nowhere to refer patients with positive SUD screenings or did not know what to do with a positive screen.

A related challenge was limited ED buprenorphine availability. Kentucky’s EDs may be more likely to initially implement screening, counseling, and education services compared to prescribing buprenorphine. The X-waiver was officially rescinded by the Substance Abuse and Mental Health Services Administration on January 12, 2023. Our survey was developed when the X-waiver was in force, and was fielded by the time the policy change occurred. Despite this landmark policy change, barriers to buprenorphine availability likely persist. Research indicates that the removal of the X-waiver had only a slight impact on the number of prescribers, indicating that workforce challenges are likely to remain relevant [24]. The main barrier to prescribing buprenorphine was a lack of provider training or willingness to prescribe, or preference to prescribe buprenorphine only in conjunction with counseling or treatment from community providers. Limited provider education, stigma, fears of diversion or legal repercussion, and limited follow-up care options were said to limit buprenorphine access.

Following KY SOS’s example, state hospital organizations can offer technical assistance in best practices for buprenorphine induction, how to implement SUD screening protocols, education about legality and limited liability of offering MOUD, and trainings for health care providers and staff alike to combat stigma of people with SUDs and MOUD. Specifically, education about medication-first approaches are needed. Information should present evidence supporting the use of MOUD regardless of counseling or treatment availability in the community, consistent with practice guidelines [25, 26]. 

Respondents were commonly concerned about improving referral pathways for people with SUD. Many participants endorsed challenges finding available treatment nearby, though access to social workers and peer support specialists were noted as helpful especially when available within the ED 24/7. Losing patients between discharge and follow-up was a recurring challenge and a deterrent from initiating patients on buprenorphine. Challenges were magnified in in smaller, rural hospitals. Respondents that identified working from small rural settings commonly noted resource constraints such as limited availability of social workers and peer support specialists, and respondents from critical access hospitals in our sample noted that expanding substance use services would be burdensome given their already limited capacity and competing priorities. A previous study of Pennsylvania hospitals found that education and addressing stigma, program champions, integration of protocols into data systems, and building relationships with community providers to facilitate warm handoffs were associated with implementation success [20]. Similarly, a study about bridge program implementation in Michigan hospitals found that social workers and peer support specialists, in particular, were key to facilitating relationships with community providers to refer patients [21]. The KY SOS program is currently providing funding to expand the peer support workforce across EDs.

Kentucky’s experience speaks to the broader challenge hospitals nationally are facing to adopt and implement SUD supports as patients with SUDs continue to present in EDs. To alleviate these challenges, policymakers could leverage opioid settlement funds or other state funding to expand staffing of peer support specialists and social workers across Kentucky’s EDs to help establish warm handoff pathways, and to expand access to naloxone. Additionally, other states can look to leverage existing relationships and infrastructure established by hospital organizations like the Kentucky Hospital Association. To fill gaps in local treatment capacity, hospital associations may also formalize methods to distribute funds for building low-threshold bridge clinics across Kentucky’s EDs. State hospital associations may also help hospitals establish referral relationships and networks with SUD supports in the community and encourage continuity of care.

### Limitations

First, responses may have been subject to self-response bias and social desirability bias, which may have led respondents to overstate the comprehensiveness of SUD services offered in their hospital EDs. Second, our study was conducted in tandem with the removal of the X-waiver requirement. As such, the context for prescribing MOUD has evolved since the time of our survey. Results reflect general perceptions of MOUD at the time the policy change occurred. Finally, this survey is cross-sectional in nature and does not speak to changes in practice and attitudes about ED SUD services over time.

## Conclusions

Our study documents reported substance use services and perceptions of implementing bridge programs across Kentucky’s emergency departments as of January to May 2023 with the goal of informing future bridge program implementation in Kentucky. We found that some KY hospitals were already implementing the full continuum of ED SUD supports, but most would need financial resources and technical assistance to offer bridge services. While some participants explicitly expressed interest in acquiring more resources and implementing bridge services, others reported having few patients in need of services or noted they did not have the capacity to implement due to competing priorities and limited staffing. Generally, EDs needed more support to have buprenorphine readily accessible for patients, integrate SUD screening into current practices, and to establish referral relationships in communities to ensure continuity of care. Kentucky’s experience may speak to the broader challenge of integrating SUD services into EDs writ large. State hospital associations, with support from state policymakers and newly available opioid settlement dollars, can mobilize technical assistance and funding support to address the challenges identified in this study.

## Electronic supplementary material

Below is the link to the electronic supplementary material.

Supplementary Material 1

## Data Availability

No datasets were generated or analysed during the current study.
