# Supplementary material for: How emergency departments are responding to the opioid crisis: Results from a statewide survey in Kentucky
Source: Addict Sci Clin Pract. 2024 Nov 8;19:78. doi: 10.1186/s13722-024-00512-3 (PMC11545227; doi:10.1186/s13722-024-00512-3)
Supplement: Supplementary file 1 — Supplementary Material 1 [file 13722_2024_512_MOESM1_ESM.docx]

**Appendices**

Appendix 1. Complete survey instrument

**Kentucky Hospital Association and Kentucky Statewide Opioid Stewardship Evaluation**

We are inviting you to participate in an electronic survey about substance use disorder services provided in the emergency department. The survey should take approximately 25 minutes to complete.

As part of this survey, we will ask you questions about substance use disorder services provided to people who you serve or interact with in the course of your work. Your answers will help KHA, KORE, and Vital Strategies to determine programming and education needs to better support emergency departments in their treatment for patients with substance use disorder across Kentucky.  While this survey is voluntary, completion is highly encouraged, as it will directly inform our programming.

Clicking "Proceed to Survey" below means that you have reviewed the information in this form and you agree to participate.

- Proceed to survey

Q1 At which hospital are you primarily employed? (Please select from the list provided. If your hospital is not listed, please contact KHA directly.)

Q2 What is your title/role at the hospital?

________________________________________________________________

For this section, we'll ask you about the availability of buprenorphine at your hospital and in the ED.

Q3 Is buprenorphine on your hospital pharmacy's formulary?

- Yes
- No
- Do not know

If Q3 = yes, respond to Q4a and Q4b:

Q4a What formulation(s) of buprenorphine are on your hospital pharmacy's formulary? (Select all that apply.)

- Suboxone (sublingual films)
- Zubsolv (sublingual tablets)
- Bunavail (buccal film)
- Sublocade (extended release)
- Do not know

Q4b How much does a buprenorphine prescription typically cost patients whose insurance does not cover the medication?

- $0
- $1-$99
- $100-$199
- $200-$299
- $300-$399
- $400-$499
- Over $500
- Do not know

Q5 Does your ED have its own pharmacy?

- Yes
- No
- Do not know

Q6 Does your ED have a Pixys Medstation system?

- Yes
- No
- Do not know

If Q6 = yes, respond to Q7:

Q7 Is buprenorphine available in the ED through Pixys?

- Yes
- No
- Do not know

Q8 Does the hospital have an outpatient pharmacy that stocks buprenorphine so patients are able to fill a buprenorphine prescription upon discharge?

- Yes
- No
- Do not know

If Q8 = yes, respond to Q9:

Q9 What hours is the outpatient hospital pharmacy open? (Select all that apply.)

- M-F during Regular Business Hours (8am - 5pm)
- M-F during Extended Hours / Evenings
- Weekends
- 24 Hours a Day / 7 Days a Week
- Other (please specify):

__________________________________________________

Q10 Rank your level of agreement with the following statement:

Patients with opioid use disorder in the ED can receive buprenorphine in a timely manner.

- Strongly agree
- Agree
- Neither agree nor disagree
- Disagree
- Strongly disagree
- Not Applicable

The next set of questions asks about your hospital's screening protocol(s) and prescribing practices for patients with a substance use disorder.

Q11 Does your ED have a screening protocol (e.g., SBIRT) to identify whether a patient has a substance use disorder?

- Yes
- No
- Do not know

If Q11 = yes, respond to Q12:

Q12 Is the screening protocol built into the electronic health record system used by the ED?

- Yes
- No
- Do not know

If Q11 = yes, respond to Q13

Q13 What screening tool(s) for substance use disorder or withdrawal does your ED regularly use? (Select all that apply.)

- Clinical Opiate Withdrawal Scale (COWS)
- Screening to Brief Intervention (S2BI) or SBIRT
- Drug Abuse Screening Test (DAST)
- Brief Screener for Alcohol, Tobacco, and Other Drugs (BSTAD)
- Tobacco, Alcohol, Prescription Medication, and other Substance Use (TAPS)
- Opioid Risk Tool (ORT)
- NIDA Quick Screen
- Alcohol Use Disorders Identification Test (AUDIT)
- Alcohol Screening and Brief Intervention for Youth (NIAAA)
- Other (please specify): __________________________________________________

If Q11 = yes, respond to Q14:

Q14 How many patients are screened for substance use disorder?

- All
- Some
- None

If Q14 = some, respond to Q15:

Q15 Because you responded that you screen some patients for substance use disorder, which of the following characteristics are included in your screening criteria? (Select all that apply.)

- Concerning PDMP findings
- Endocarditis
- Hepatitis C
- Opioid prescription
- Overdose
- Skin infections/abscesses
- Adolescents
- Older adults
- Pregnant
- Other (please specify): __________________________________________________

Q16 What barriers does your ED face related to screening patients for substance use disorder? (Select all that apply.)

- Lack of time
- Need to triage competing medical problems
- Lack of clinical knowledge/training in administering substance use disorder screening
- Lack of training in what to do with a positive screen
- Nowhere to refer patients with a positive screen
- Some staff are uncomfortable screening patients for substance use disorder
- Lack of adequate substance use disorder screening tools
- Patient privacy concerns (e.g., family member or significant other will not leave the room)
- Screening is not embedded within the EMR
- Screening patients for substance use disorder is not part of the ED protocol
- Other (please specify): __________________________________________________

If Q11 = yes, respond to Q17:

Q17 What factors make it easier to screen patients for substance use disorder? (Select all that apply.)

- Substance use disorder screening is embedded in the EMR
- Substance use disorder screening is part of the ED protocol
- ED has a champion who has led education efforts about screening for substance use disorder
- Providers have clinical knowledge/training in administering substance use disorder screening
- Providers are trained in what to do with a positive screen
- Providers know how/where to refer patients with a positive screen
- Providers are comfortable administering substance use disorder screenings
- Other (please specify): _____________________________________________

If Q11 = yes, respond to Q18a:

Q18a Does your ED have any plans to modify screening processes for substance use disorder?

- Yes
- No
- Do not know

If Q18a = yes, respond to Q18b:

Q18b Please describe your plans to modify the screening processes for substance use disorder.

________________________________________________________________

________________________________________________________________

________________________________________________________________

If Q11 = no, respond to Q19a:

Q19a Does your ED have any plans to implement screening processes for substance use disorder?

- Yes
- No
- Do not know

If Q19a = yes, respond to Q19b:

Q19b Please describe your plans to implement screening processes for substance use disorder.

________________________________________________________________

________________________________________________________________

________________________________________________________________

For this set of questions, we'll ask about the buprenorphine prescribing practices in the ED, availability of X-waivered providers, and referrals.

Q20 Does your ED have a written protocol to prescribe buprenorphine to patients with opioid use disorder?

- Yes
- No
- Do not know

If Q20 = yes, respond to Q21:

Q21 What is the approximate date this was implemented? (MM/DD/YYYY)

________________________________________________________________

If Q20 = yes, respond to Q22:

Q22 Please describe the protocol to prescribe buprenorphine to patients with opioid use disorder.

________________________________________________________________

________________________________________________________________

________________________________________________________________

|  |
| --- |

Q23 Does the ED have physicians, PAs, or APRNs who dispense buprenorphine using the 3-day exemption rule instead of under an X-waiver?

*(The 3-day exemption rule allows clinicians to prescribe buprenorphine without an X-waiver under Title 21, Code of Federal Regulations, Part 1306.07(b). Clinicians in the ED are allowed to dispense methadone and buprenorphine for opioid use disorder without an opioid treatment program license, for 3 days.)*

- Yes
- No
- Do not know

Q24 Approximately what proportion of prescribers in your ED are aware of the 3-day exemption rule that allows them to prescribe buprenorphine for opioid use disorder in the ed without an X-waiver?

- None
- 1% - 25%
- 26% - 50%
- 51% - 75%
- Over 75%
- Do not know

If Q23 = yes, respond to Q25:

Q25 Approximately what proportion of your ED clinicians induct patients on buprenorphine in this way?

- None
- 1% - 25%
- 26% - 50%
- 51% - 75%
- Over 75%
- Do not know

If Q23 = yes, respond to Q26:

Q26 Approximately what percent of patients inducted on buprenorphine in your ED return for doses on Days 2 or 3?

- Less than 25%
- 26% - 50%
- 51% - 75%
- Over 75%
- Do not know

Q27 Approximately how many physicians, PAs, and APRNs are on staff in your ED? Include all FTE and non-FTEs in the ED.

- Physicians __________________________________________________
- Physician Assistants __________________________________________________
- APRNs/Nurse Practitioners ________________________________________________

Q28 Approximately what number of providers in your ED have an X-waiver to prescribe buprenorphine? Include all FTE and non-FTEs in the ED.

- Physicians __________________________________________________
- Physician Assistants __________________________________________________
- APRNs/Nurse Practitioners __________________________________________________

Q29 Does the ED facilitate a home induction option, where providers with an X-waiver prescribe buprenorphine for patients to take home once their opioid withdrawal is sufficiently resolved?

- Yes
- No
- Do not know

If Q29 = yes, respond to Q30:

Q30 Approximately how many X-waivered providers prescribe take-home buprenorphine prescriptions?

- None
- 1%- 25%
- 26% - 50%
- 51% - 75%
- Over 75%
- Do not know

If Q29 = yes, respond to Q31:

Q31 Approximately what percent of patients with opioid use disorder receive take-home buprenorphine?

- Less than 25%
- 26% - 50%
- 51% -75%
- Over 75%
- Do not know

Q32 What barriers are there to prescribing take-home buprenorphine? (Select all that apply.)

- Pharmacy does not stock buprenorphine or maintain adequate supplies
- Limited access to pharmacy or long wait times
- Lack of providers that have an X-waiver to prescribe buprenorphine
- Lack of patient interest
- Lack of clinician willingness to prescribe buprenorphine
- Lack of clinician knowledge in how to induct patients on buprenorphine
- No community providers to continue prescriptions after take-home supply runs out
- Clinicians often will not prescribe buprenorphine unless patients are connected to counseling or treatment
- Lack of time to follow up with patient when they leave the ED
- Lack of knowledge that patients can receive take-home buprenorphine from X-waivered providers
- Other (please specify): ____________________________________________

Q33 Of the X-waivered providers in your ED, approximately how many have prescribed buprenorphine within the past 12 months?

- None
- 1% - 25%
- 26% - 50%
- 51% - 75%
- Over 75%
- Do not know

Q34 What are the top barriers your hospital faces in getting ED providers to obtain an X-waiver to prescribe buprenorphine?

- Lack of time to obtain waiver
- Lack of interest in prescribing buprenorphine
- Lack of incentive to obtain waiver
- Lack of knowledge in how to treat opioid use disorder
- Lack of need to obtain waiver with the 3-day rule
- Other (please specify):

Q35 Which of the following factors have encouraged ED providers to become X-waivered to prescribe buprenorphine?

- Champion in the ED or hospital who helps providers obtain waivers
- ED/hospital trains providers to treat patients with opioid use disorder
- ED/hospital provides incentives to obtain a waiver
- More providers can obtain waivers now (NPs, PAs)
- DEAs removal of the training requirement to treat up to 30 patients
- Other (please specify): _____________________________________________

Q36 Does the ED provide any non-medication interventions (e.g., counseling, motivational interviewing, education) for patients with opioid use disorder?

- Yes
- No
- Do not know

If Q36 = yes, respond to Q37:

Q37 Please describe any non-medication interventions (e.g., counseling, motivational interviewing, education) for patients with opioid use disorder.

________________________________________________________________

________________________________________________________________

________________________________________________________________

Q38 Are any non-medication interventions (e.g., counseling) for patients with opioid use disorder required for a patient to receive medications for opioid use disorder?

- Yes
- No
- Do not know

If Q38 = yes, respond to Q39:

Q39 Please describe the non-medication interventions (e.g., counseling) required for a patient to receive medications for opioid use disorder.

________________________________________________________________

________________________________________________________________

________________________________________________________________

Q40 Is there an existing bridge clinic in or near the hospital that the ED uses to refer patients?

- Yes
- No
- Do not know

.

Q41 Below is a list of services for follow-up care.

Please indicate to which ones your ED refers patients, if any. (Select all that apply.)

- Step down care within the hospital
- Primary care provider
- Behavioral health provider (e.g., psychiatrist, licensed counselor, etc.)
- Inpatient substance use disorder treatment
- Outpatient substance use disorder treatment
- Residential substance use disorder treatment
- Opioid treatment program (methadone treatment)
- Buprenorphine treatment
- No referrals process in place
- Other (please specify): _____________________________________________

Q42 What are the top barriers to making warm hand-offs to other providers for follow-up care? (Select all that apply.)

- No partnerships with existing providers
- No providers nearby
- No providers who accept Medicaid as payment
- No providers with availability for new patients
- Lack of time to coordinate
- Lack of staff who can coordinate hand-offs
- Providers do not want to onboard patients who are in crisis
- Patients not interested in hand-offs
- Our ED currently does not have a protocol for referrals
- Other (please specify): _____________________________________________

The next set of questions is about the use of peer support specialists and coaches for patients with substance use disorder.

Q43 Does the hospital utilize peer support specialists or coaches for patients with substance use disorder in the ED?

- Yes
- No
- Do not know

If Q43 = yes, respond to Q44:

Q44 Please describe the use of peer support specialists or coaches.

________________________________________________________________

________________________________________________________________

________________________________________________________________

If Q43 = yes, respond to Q45:

Q45 On a scale of 1-10 (1 being "Not at all important" and 10 being "Extremely important"), how important are peer support specialists and/or coaches to improving patient outcomes in patients with substance use disorder?

|  | 1  (Not at all important) | 2 | 3 | 4 | 5 | 6 | 7 | 8 | 9 | 10 (Extremely important) |
| --- | --- | --- | --- | --- | --- | --- | --- | --- | --- | --- |
| Importance |  |  |  |  |  |  |  |  |  |  |

If Q43 = yes, respond to Q46:

Q46 When are peer support specialists and/or coaches available? (Select all that apply.)

- Normal business hours (e.g., 8am-5pm)
- Evening
- Overnight
- Weekends
- Other (please specify): ____________________________________________

Q47 Does your ED have plans to implement or expand a peer support specialist program in your ED?

- Yes
- No
- Do not know

If Q47 = yes, respond to Q48:

Q48 Please describe your plans to implement or expand the use of peer support specialists.

________________________________________________________________

________________________________________________________________

________________________________________________________________

If Q47 = yes, respond to Q49:

Q49 Upon implementation or expansion of your peer support specialist program, when would peer support specialists be available? (Select all that apply.)

- Normal business hours (e.g., 8am-5pm)
- Evening
- Overnight
- Weekends
- Other (please specify): ___________________________________________

Now we want to know about access to social services among patients with substance use disorder in your ED.

Q50 Does the ED have direct access to hospital and/or ED social workers for patients with substance use disorder?

- Yes
- No
- Do not know

If Q50 = yes, respond to Q51:

Q51 Please describe the ED's access to social workers for patients with substance use disorder.

________________________________________________________________

________________________________________________________________

________________________________________________________________

Q52 Which of the following social services does the ED provide for patients with substance use disorder? (Select all that apply.)

- Assistance with transportation
- Housing resources
- Assistance navigating insurance benefits
- Documentation (e.g. ID card)
- Assistance obtaining Medicaid or other health coverage
- Other (please specify): _____________________________________________
- None of the above

If any social services are selected in Q52, respond to Q53:

Q53 Who provides these social services? (Select all that apply.)

- ED social worker
- Community partner
- Peer support specialist
- Other (please specify): _____________________________________________

Q54 Which of the following social services does the ED refer patients with substance use disorder? (Select all that apply.)

- Assistance with transportation
- Housing services
- Assistance navigating insurance benefits
- Documentation (e.g., ID card)
- Assistance obtaining Medicaid or other health coverage
- Other (please specify): ____________________________________________
- None of the above

Q55 What barriers does the ED face in providing services or referrals for social services? (Select all that apply.)

- Lack of partnerships with existing service providers
- Lack of service providers nearby
- Lack of service providers with availability for new clients
- Lack of time to coordinate services or referrals
- Lack of staff to coordinate services or referrals
- ED does not have the capacity to contact patients after discharge to ensure care continuity
- Patients are not interested in services or referrals
- Other (please specify): ____________________________________________

Q56 What factors help your ED provide services or referrals for social services?

- ED has a champion for connections to social services
- ED has social services navigators on staff
- ED has existing partnerships with social service providers
- ED has follow-up care staff who contact patients after discharge to ensure care continuity
- Other (please specify): _____________________________________________

The next set of questions ask about harm reduction practices and opioids commonly used in the ED.

Q57 Which of the following harm reduction services are standard protocol in your ED? (Select all that apply.)

- Overdose education
- Take-home naloxone
- Co-prescribing naloxone with opioid prescriptions
- Safer use education
- Safer use supplies such as fentanyl test strips
- Wound care kit
- No harm reduction services provided
- Other (please specify): _____________________________________________

If in Q57, the option “Take-home Naloxone” is not selected, respond to Q58:

Q58 Does your ED have any plans to implement a Naloxone take-home protocol?

- Yes
- No
- Do not know

If Q58 = yes, respond to Q59:

Q59 Please describe your plans to implement a Naloxone take-home protocol in the ED.

________________________________________________________________

________________________________________________________________

________________________________________________________________

Q60 Which of the following harm reduction services does your ED refer to community partners? (Select all that apply.)

- Take-home naloxone (community RX, mobile naloxone unit, etc.)
- Local health department (safer use discussion/education, wound care kit, etc.)
- Syringe access services (needle exchange program)
- Other (please specify): _____________________________________________
- No referrals are made for harm reduction

Q61 Which parenteral opioid(s) are most commonly utilized in your ED? (Select all that apply.)

- Morphine
- Hydromorphone
- Fentanyl
- Other (please specify): _____________________________________________

Q62 Which oral opioid(s) are most commonly utilized in your ED? (Select all that apply.)

- Oxycodone
- Hydrocodone
- Tramadol
- Other (please specify): ________________________________________

Q63 What is the default pill count (# to dispense) for the most common opioid prescriptions?

- Less than 3 days
- 3-5 days
- 5-10 days
- Other (please specify): __________________________________________________

In the following section, we want to know about training to address stigma, cultural competency, and racial bias, as well as policies to improve health equity.

Q64 Have any of the ED staff participated in any form of stigma reduction training (training focused on reducing negative attitudes towards individuals with substance use disorder)?

- Yes
- No
- Do not know

If Q64 = yes, respond to Q65:

Q65 Please describe this stigma reduction training and staff participation.

________________________________________________________________

________________________________________________________________

________________________________________________________________

Q66 Does your ED have plans to implement or expand stigma reduction training for ED staff?

- Yes
- No
- Do not know

If Q66 = yes, respond to Q67:

Q67 Please describe your plans to implement or expand stigma reduction training for ED staff.

________________________________________________________________

________________________________________________________________

________________________________________________________________

Q68 Have any of the ED staff participated in any form of racial equity or cultural competency training (training focused on addressing and reducing the negative attitudes and implicit bias towards Black, Indigenous, and People of Color (BIPOC) individuals)?

- Yes
- No
- Do not know

If Q68 = yes, respond to Q69:

Q69 Please describe the racial equity and/or cultural competency training and staff participation.

________________________________________________________________

________________________________________________________________

________________________________________________________________

Q70 Does your ED have plans to implement or expand racial equity training for ED staff?

- Yes
- No
- Do not know

If Q70 = yes, respond to Q71:

Q71 Please describe your plans to implement or expand racial equity training.

________________________________________________________________

________________________________________________________________

________________________________________________________________

Q72 What are your ED's barriers to reducing stigma and racial bias for patients with substance use disorder?

- Lack of time to complete training in these areas
- Lack of staff interest
- Competing training interests
- No CME eligible trainings on these topics available
- Other (please specify): ____________________________________________
- Not applicable

Q73 Are there barriers for Black, Indigenous, and People of Color (BIPOC) individuals with substance use disorder to be able to access medications for opioid use disorder in the ED?

- Yes
- No
- Do not know

If Q73 = yes, respond to Q74:

Q74 Please describe the barriers for Black, Indigenous, and People of Color (BIPOC) individuals with substance use disorder to be able to access ED and/or MOUD care.

________________________________________________________________

________________________________________________________________

________________________________________________________________

Q75 Does your hospital have policies or interventions to help produce racially equitable outcomes for patients with substance used disorder at your hospital?

- Yes
- No
- Do not know

If Q75 = yes, respond to Q76:

Q76 Please describe your hospital’s policies or interventions to help produce racially equitable outcomes for patients with substance use disorder.

________________________________________________________________

________________________________________________________________

________________________________________________________________

The next set of questions asks about providers in the community and any displaced patients with opioid use disorder.

Q77 If a provider in the community loses DEA prescribing rights abruptly, who is the best person to notify in the ED of patients possibly seeking treatment/prescriptions until established with another provider in the community?

________________________________________________________________

________________________________________________________________

________________________________________________________________

Q78 What is the best way to notify the ED and providers under these circumstances?

________________________________________________________________

________________________________________________________________

________________________________________________________________

Q79 What information must you know to efficiently and thoroughly treat these patients?

________________________________________________________________

________________________________________________________________

________________________________________________________________

Q80 Would you prefer a standardized communication process when dealing with this specific situation?

- Yes
- No
- Do not know

If Q80 = yes, respond to Q81:

Q81 Please describe what would be most helpful to include in a standardized communication process.

________________________________________________________________

________________________________________________________________

________________________________________________________________

Lastly, we want to know what services are the most challenging to implement and what services are the most important for your ED implement to treat people with substance use disorder.

Q82 Of the items listed below, please indicate which two are the most challenging for your ED to implement.

- Naloxone dispensing
- Screening for substance use disorder
- Increasing x-waivered providers
- Buprenorphine induction in the ED
- Counseling and education
- Referring to community-based providers
- Peer support specialists
- Social work services
- Harm reduction
- ED staff stigma reduction
- Other (please specify): _____________________________________________

Q83 Please explain your two choices.

________________________________________________________________

________________________________________________________________

________________________________________________________________

Q84 Of the items listed below, please indicate which two are the most important for your ED to implement.

- Naloxone dispensing
- Screening for substance use disorder
- Increasing x-waivered providers
- Buprenorphine induction in the ED
- Counseling and education
- Referring to community-based providers
- Peer support specialists
- Social work services
- Harm reduction
- ED staff stigma reduction
- Other (please specify): _____________________________________________

Q85 Please explain your two choices.

________________________________________________________________

________________________________________________________________

________________________________________________________________

Q86 Is there anything else you want our team to know?

________________________________________________________________

________________________________________________________________

________________________________________________________________

Q87 Thank you for completing the survey. Use the back button below to review your survey responses.  If you are ready to submit, select "Submit Survey Response" below.

- Submit Survey Response
